# Post-radiotherapy Thyroid Cartilage Chondroradionecrosis

**DOI:** 10.7759/cureus.87209

**Published:** 2025-07-03

**Authors:** Modi A AlKharfi, Rayyan A AlQurayyan, Shahad H AlRaddadi, Mohammed A AlSaif, Mohammed S Wadani, Fahad N Al-Ghamdi

**Affiliations:** 1 Radiology, King Fahad Medical City, Riyadh, SAU

**Keywords:** chondroradionecrosis, dysphagia, hoarseness, ionizing radiation, laryngeal cartilage, thyroid cartilage

## Abstract

Radiotherapy is a widely used treatment modality for various cancers, particularly head and neck malignancies. While effective, it can result in a range of adverse effects and complications that impact the patient's quality of life. Chondroradionecrosis (CRN) has been identified as a significant concern, particularly affecting the laryngeal cartilage.

We present the case of a 55-year-old male diagnosed with poorly differentiated cancer of the left thyroid lobe with areas of anaplastic differentiation. He underwent total thyroidectomy in April 2021, followed by external beam radiation therapy (70 Gray in 35 fractions), which was completed in August 2021. The patient presented to the emergency department in August 2024 with complaints of neck swelling, dysphagia, and hoarseness for the past month, which had worsened over the previous 10 days. Radiological studies revealed a destructive osseous process involving the right lamina of the thyroid cartilage, characterized by cortical fragmentation and intraosseous air bubbles, accompanied by edematous changes in both vocal cords, resulting in subsequent airway narrowing.

CRN can lead to substantial morbidity, including cartilage fragmentation, collapse, and potential loss of function. In this case study, we aim to explore the clinical manifestations and imaging findings of CRN involving the thyroid cartilage in patients undergoing radiotherapy.

## Introduction

Radiation therapy is a widely employed oncologic treatment modality that uses ionizing radiation to control or eliminate malignant cells. It plays a crucial role in the management of head and neck cancers and is also used as an adjuvant therapy for various other malignancies. While radiation therapy offers significant therapeutic benefits, it is associated with several potential complications. One such complication is chondroradionecrosis (CRN), a condition characterized by the degeneration and destruction of cartilage due to radiation exposure. This complication most commonly affects the laryngeal cartilage, often manifesting as fragmentation or collapse of the affected area. CRN is a rare and serious complication of radiotherapy that can be fatal if not managed aggressively [1].

Necrosis may be associated with the formation of a fistula in the overlying skin and persistent nonhealing for more than three months when radiation doses exceed 60 Gy [2].

In this case report, we describe a patient with a history of thyroid cancer who underwent total thyroidectomy followed by radiation therapy. During subsequent follow-ups, imaging findings indicative of CRN specifically involving the thyroid cartilage were observed. This report aims to discuss the clinical manifestations and imaging features of CRN affecting the thyroid cartilage in patients who have undergone radiation therapy.

## Case presentation

**Figure 1:**
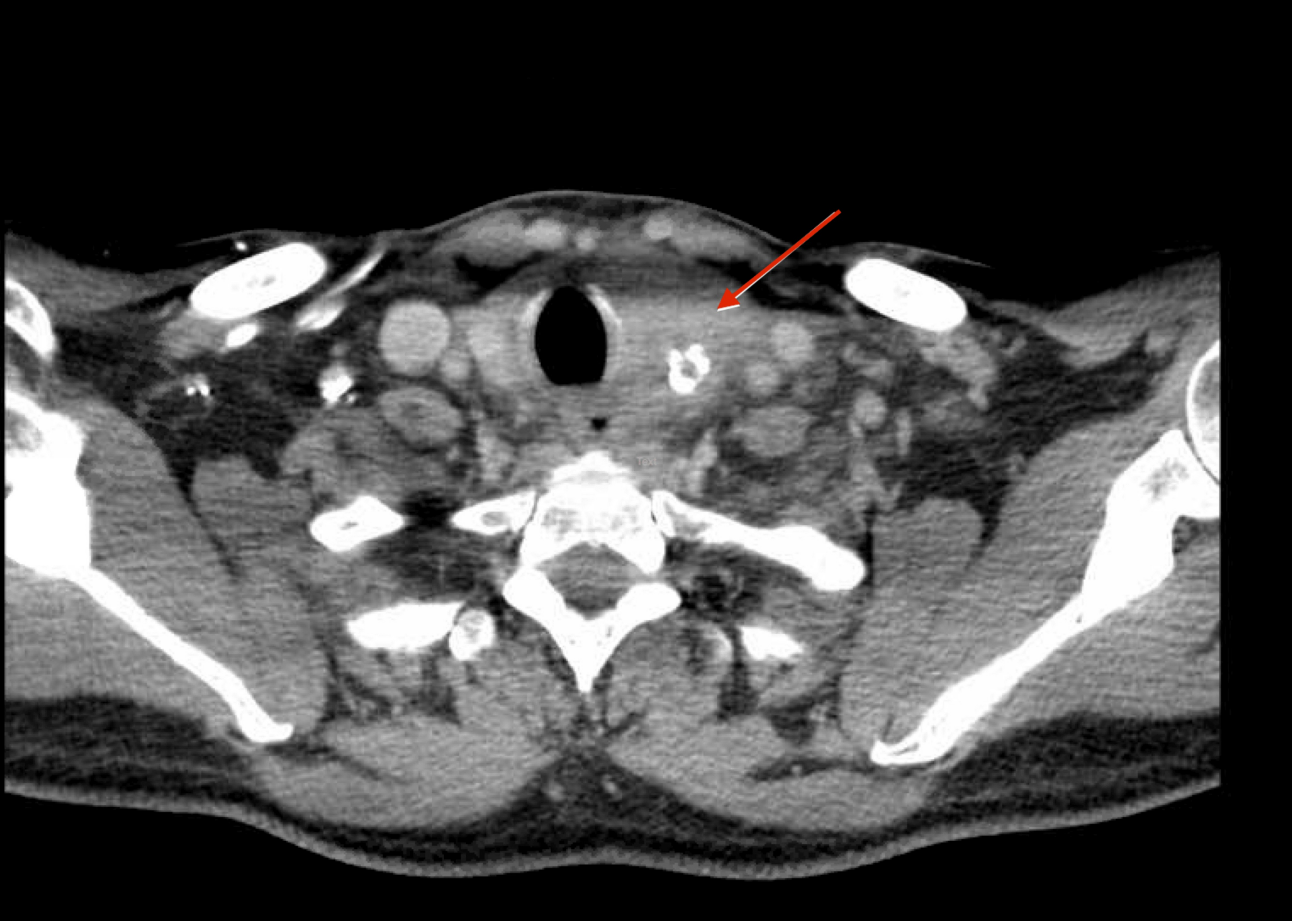
Large heterogeneous mass with calcifications in the left thyroid lobe

This is a case of a 58-year-old male who presented to the emergency department at the age of 55 in March 2021, complaining of left neck swelling that had worsened over the past week, with pain radiating to the left ear and associated dysphagia to solids, odynophagia, and hoarseness. A head and neck CT scan revealed a large, heterogeneous mass with calcification and adjacent fat stranding replacing the left thyroid lobe, measuring 4 × 4.6 × 6.2 cm (anteroposterior, transverse, and coronal, respectively) (Figure 1). It was associated with suspicious regional left cervical lymph nodes, the largest of which was located at level III and measured 1.3 cm. The head and neck CT was followed by fine-needle aspiration (FNA), which revealed a poorly differentiated thyroid cancer with papillary tall cells and anaplastic components. The patient was treated with total thyroidectomy and bilateral neck dissection on 27 April 2021, followed by external beam radiation therapy, which was completed on 9 August 2021.

**Figure 2:**
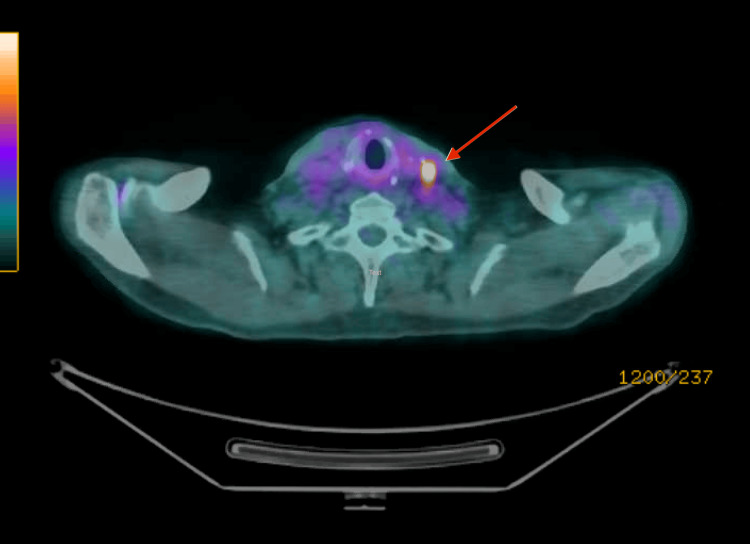
FDG-avid cervical lymphadenopathy at left level III FDG: fluorodeoxyglucose

During regular follow-up, a PET scan performed on 28 September 2022 revealed ongoing fluorodeoxyglucose (FDG)-avid left level III cervical lymphadenopathy (Figure 2). FNA was subsequently performed, and the result indicated metastatic poorly differentiated carcinoma. He began treatment with lenvatinib in November 2022 but was switched to cabozantinib in November 2023 due to disease progression. However, the patient has been poorly adherent to his medication.

**Figure 3:**
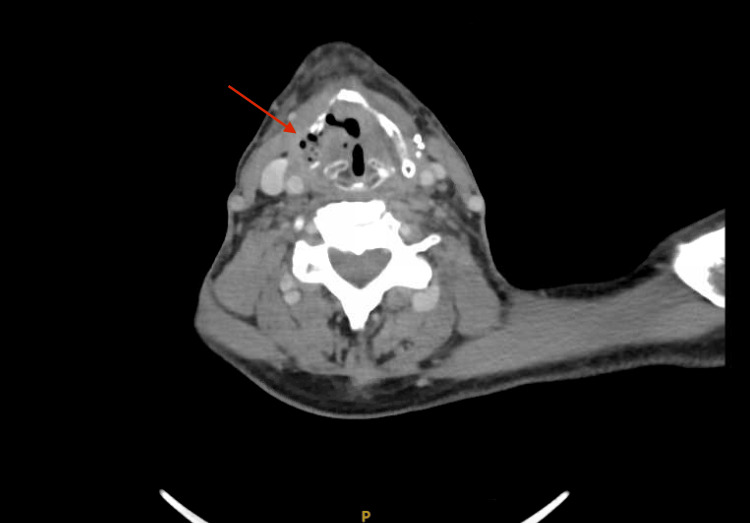
Destructive osseous process involving the right lamina of the thyroid cartilage

On 6 August 2024, the patient presented to the emergency department complaining of new-onset neck swelling, dysphagia, and hoarseness for the past month, which had worsened over the previous 10 days. A CT scan of the head and neck was performed, showing interval development of a destructive osseous process involving the right lamina of the thyroid cartilage. The findings were suggestive of CRN (Figure 3).

On 27 August 2024, the patient presented to the emergency department again, this time complaining of an increase in the size of the neck swelling, now associated with an open fistula in the anterior neck with purulent discharge for the past three days. The patient reported that dysphagia had progressively worsened and that he could barely swallow water without experiencing severe pain and difficulty. This was also associated with coughing and phlegm that appeared coffee-colored. Additionally, his family reported a weight loss of 7 kg over the past few months along with a loss of appetite.

**Figure 4:**
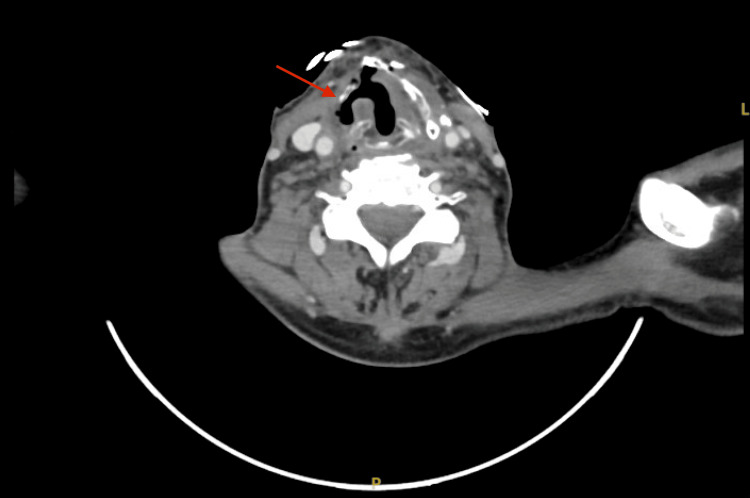
CRN communicating with a small laryngocele CRN: chondroradionecrosis

**Figure 5:**
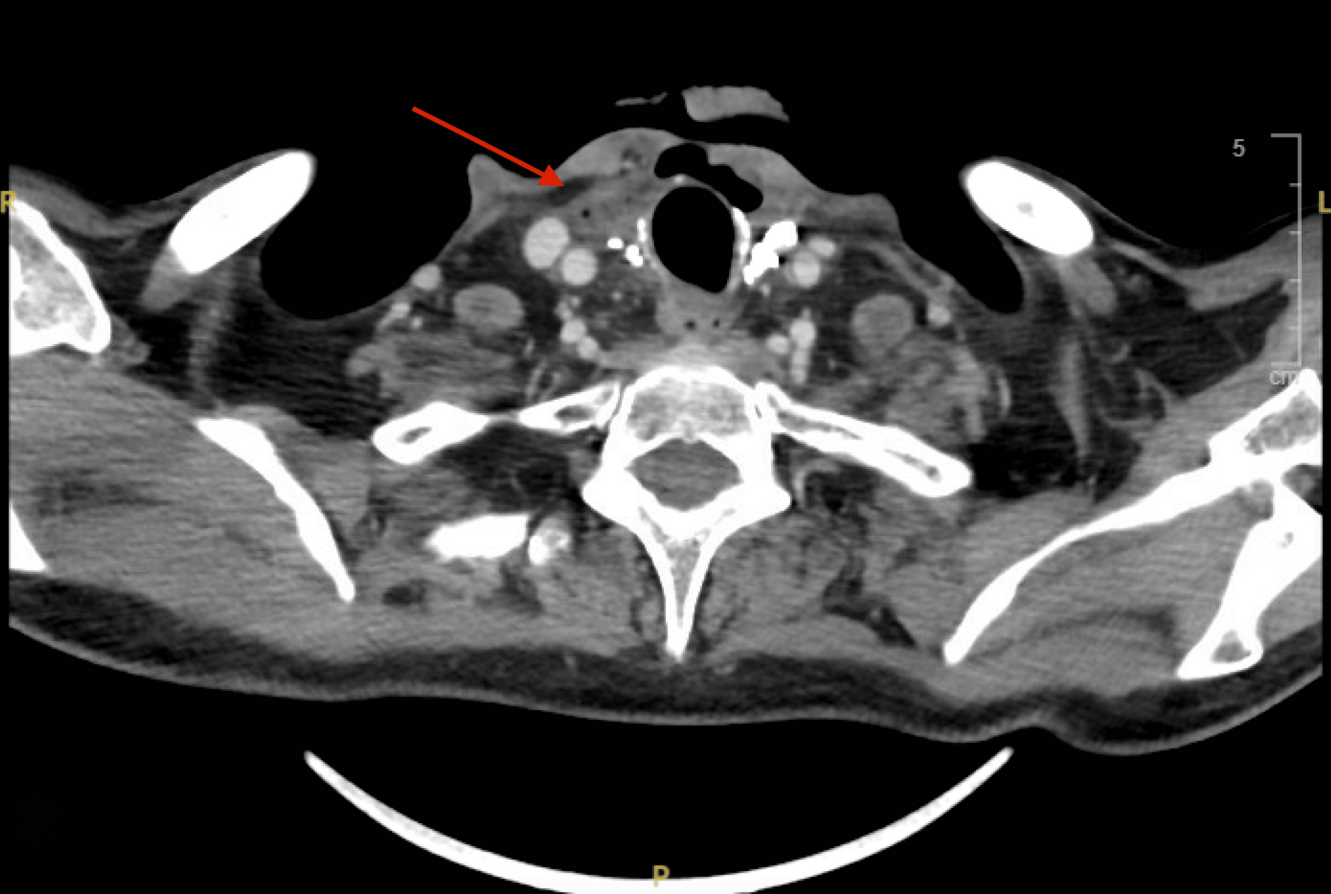
New development of a small gas-forming loculated collection within the right thyroid bed extending to the anterior neck, suggestive of superadded infection

A CT of the head and neck performed on 28 August 2024 showed a stable appearance of an osseous process involving the right lamina of the thyroid cartilage, which may suggest CRN communicating with a small laryngocele (Figure 4), associated with the new development of a small gas-forming loculated collection within the right thyroid bed extending to the anterior neck (Figure 5), suggestive of a superadded infection.

## Discussion

The presented case report highlights the occurrence of one of the late adverse effects of radiotherapy. As is well known, the adverse effects of radiation are classified into early (occurring within 90 days) and late (occurring more than 90 days) reactions based on the time of onset. Late effects of radiotherapy include osteoradionecrosis, CRN, radiation-induced vasculopathy, carotid blowout syndrome, and radiation-induced malignancies [3].

This report focuses on CRN of the thyroid cartilage, shedding light on its clinical manifestations and imaging features in patients who have undergone radiation therapy.

CRN is a rare complication, typically presenting with laryngeal deformity. In this particular case, CRN was identified in the thyroid cartilage (Figure 3). Confirming the diagnosis proved challenging, as the patient passed away before follow-up. The patient, a known case of thyroid cancer, had undergone total thyroidectomy followed by radiation therapy. He presented years later with complaints of neck swelling, hoarseness, and dysphagia. The dysphagia had progressed to the point where he could barely swallow water without severe pain and difficulty. He also reported coughing up coffee-colored phlegm, and his family noted a weight loss of 7 kg over the past few months. A fistula and pus discharge developed from the neck swelling after a few days.

The above-described symptoms are similar to those observed in laryngeal CRN, which include odynophagia, dysphonia, and arytenoid edema. These symptoms support CRN as a relevant differential diagnosis [4].

Given that clinical examination alone may not be sufficient for a definitive diagnosis, imaging studies such as CT scans or MRI provide valuable insights into the characteristics of thyroid cartilage CRN. However, PET/CT imaging can sometimes be misleading in cases involving the head and neck. CRN typically peaks within the first 12 months following radiation therapy but may also occur years later.

In terms of management, symptoms can be classified into four grades. Grades 1 and 2 (early stages) include hoarseness and dysphagia and are usually treated with conservative measures such as humidified oxygen, steroids, and anti-reflux medications. Grade 3 represents a more severe but uncomplicated disease and may require systemic steroids, culture-directed antibiotics, and hyperbaric oxygen therapy if symptoms are resistant. If conservative management fails, aggressive surgical intervention may be needed. Grade 4 involves life-threatening complications such as severe stridor indicating airway obstruction, carotid artery blowout, extensive soft tissue necrosis, and local or systemic sepsis. In such cases, hospital admission is necessary for close monitoring, intravenous antibiotics, and possibly a tracheotomy if the airway is compromised. Total laryngectomy may also be considered, even in the absence of a conclusive biopsy [4,5].

## Conclusions

This case report highlights the importance of recognizing one of the late complications of radiotherapy: cartilage CRN. The case highlights the diagnostic challenges and underscores the need for a multidisciplinary approach that incorporates clinical examination, imaging studies, and clinical manifestations. Furthermore, it highlights the importance of individualized treatment plans tailored to the specific characteristics and extent of CRN. Conservative management, including antibiotics for infections induced by CRN or hyperbaric oxygen therapy, may be considered in some cases. In contrast, severe cartilage damage often requires resection. Continued research and reporting of such cases will contribute to a better understanding of this rare complication and aid in developing optimal management strategies.
